# Construction of a pneumolysin deficient mutant in streptococcus pneumoniae serotype 1 strain 519/43 and phenotypic characterisation

**DOI:** 10.1016/j.micpath.2020.103999

**Published:** 2020-04

**Authors:** Vanessa S. Terra, Charles D. Plumptre, Emma C. Wall, Jeremy S. Brown, Brendan W. Wren

**Affiliations:** a Department of Infection Biology, Faculty of Infectious and Tropical Diseases, London School of Hygiene and Tropical Medicine, London, WC1E 7HT, United Kingdom; b Centre for Inflammation and Tissue Repair, Department of Medicine, Royal Free and University College Medical School, Rayne Institute, London, WC1E 6JF, United Kingdom; c Division of Infection and Immunity, UCL Cruciform Building, London, WC1E 6BT, United Kingdom

**Keywords:** *S. pneumoniae*, Mutagenesis, Serotype 1, Pneumolysin, Haemolysis

## Abstract

*Streptococcus pneumoniae* capsular serotype 1 continues to pose a huge infectious disease burden in low- and middle-income countries, particularly in West Africa. However, studies on this important serotype have been hampered by the inability to genetically modify these strains. In this study we have genetically modified a serotype 1 strain (519/43), the first time that this has been achieved for this serotype, providing the methodology for a deeper understanding of its biology and pathogenicity. As proof of principle we constructed a defined pneumolysin mutant and showed that it lost its ability to lyse red blood cells. We also showed that when mice were infected intranasally with the mutant 519/43Δ*ply* there was no significant difference between the load of bacteria in lungs and blood when compared to the wild type 519/43. When mice were infected intraperitoneally there were significantly fewer bacteria recovered from blood for the mutant 519/43Δ*ply* strain, although all mice still displayed signs of disease. Our study demonstrates *S. pneumoniae* serotype 1 strains can be genetically manipulated using our methodology and demonstrate that the ability to cause pneumonia in mice is independent of active pneumolysin for the 519/43 serotype 1 strain.

## Highlights

• Mutagenesis in Serotype 1 *S. pneumoniae* is possible in strain 519/43.
• 519/43 possess pneumolysin D380 N, however it is not more haemolytic than the pneumolysin present in D39.
• 519/43 strain is capable of causing disease independently of pneumolysin.

## 1 Introduction

*Streptococcus pneumoniae* (*S. pneumoniae*, the pneumococcus) is a major cause of morbidity and mortality worldwide. To date, nearly 100 serotypes of *S. pneumoniae* have been discovered [[1], [2], [3], [4], [5], [6], [7]]. Invasive pneumococcal disease (IPD) is responsible for nearly 1 million deaths per year in children under the age of 5 [8] with S. *pneumoniae* being the leading cause of bacterial pneumonia, otitis media, meningitis and septicaemia worldwide [9]. Over 90% of the pneumococcal burden is in low income countries predominantly in sub-Saharan Africa, Latin America and Asia [[10], [11], [12]]. Serotype 1 has been responsible for outbreaks particularly in the African meningitis belt, where the highly virulent sequence type ST217 is predominant [[13], [14], [15], [16], [17], [18]]. Gessner et al. showed that serotype 1 *S. pneumoniae* was as important as *Neisseria meningitidis* for incidence and mortality of bacterial meningitis in the African Meningitis Belt [19]. However, despite being found in invasive disease often, this serotype is rarely found in carriage. For example, in The Gambia, serotype 1 *S. pneumoniae* is responsible for about 20% of all invasive disease cases and is only found in about 0.5% of healthy carriers [17,[20], [21], [22]]. Serotype 1 has be shown to be carried for approximately 9 days, being the second shortest carriage rate described amongst pneumococci [23]. Competent pneumococci undergo genetic exchange and recombination more efficiently in carriage rather than invasive disease [24]. Since serotype 1 is rarely found in carriage the hypothesis that it might have a lower recombination rate has been proposed [25]. Recently, it has been reported that serotype 1 was disseminated within the African continent but it diversified by country, most likely due to population specific pressures, leading to stable circulation of clones within its own geographic regions [26,27]. Sampane-Donkor et al., reported that the current serotype 1 circulating sequence types in West Africa are ST618, and ST612, ST3579, ST3960, ST3965 which are all variations of ST618. Most recently, Chaguza et al. have added that the highly virulent sequence type ST217 has spread to different countries and continents [28]. To define why serotype 1 strains are dominant causes of invasive disease in sub-Saharan Africa despite their relative rarity in carriage a systematic analysis of mutated genes in representative strains is required. However until now, all attempts of mutagenesis in serotype 1 have been unsuccessful [29]. In this study we report the mutagenesis of a serotype 1 strain, 519/43, that is capable of acquiring and recombining new DNA into its genome. In order to demonstrate that mutations in this background are possible, a pneumolysin deficient mutant was constructed, verified and characterised phenotypically *in vitro* and *in vivo.* Being able to investigate the effects of mutating specific genes will allow a deeper understanding why serotype 1 *S. pneumoniae* strains are dominant causes of meningitis in sub-Saharan Africa.

## 2 Methods

### 2.1 Bacterial strains and growth conditions

*Streptococcus pneumoniae* serotype 2 strain D39 and serotype 1 strain 519/43 S T 5316 [30], isolated in 1943 in Denmark, acquired from the Statens Serum Institute, were used in this study. Consistently, pneumococci were grown in brain heart infusion (BHI) broth or on blood agar plates supplemented with 5% (vol/vol) defibrinated horse blood under microaerophilic conditions at 37 °C. Where appropriate, spectinomycin (100 μg/ml) was added to the culture medium.

**Mutagenesis overview:** The method used in this study starts by using PCR-SOE to eliminate an important part of the gene of interest and to introduce a restriction digestion site. In this case a 191 bp from the middle of the *ply* gene was deleted and a BamHI site introduced. This fragment was then cloned in pGEMTeasy using TA cloning to generate pSD1 (Fig. 2). This plasmid was then digested at the newly introduced BamHI site and ligated to a spectinomycin cassette termed pSD2 (Fig. 4A). pSD2 was then used to transform 519/43WT, acting as a suicide plasmid.

### 2.2 Splicing by overlapping extension PCR (SOE-PCR) [31] and amplification of the spectinomycin cassette

PCR for the amplification of the upstream (488 bp) and downstream (715 bp) homology arms from the flanking regions of the *ply* gene from 519/43 strain (serotype 1) was performed using plyFw1_ NotI, plyRv1_BamHI, plyFw2_BamHI and plyRv2_NotI respectively (Table 1). Primers used for the homologous arms for SOE_PCR were designed using the D39 genome (NC_003098/NC_008533). The products were analysed by gel electrophoresis and excised from the gel, followed by purification according to the manufacturer's instructions (Monarch Gel Extraction, NEB, UK). Next using equimolar amounts of upstream and downstream homology arms as templates, these two fragments were fused together by SOE PCR using primers plyFw1_ NotI, and plyRv2_NotI. At the time of the amplification relevant restriction sites were introduced, BamHI at the SOEing site for later ligation of the spectinomycin cassette, as well as, NotI sites at the 5′ and 3′ of the PCR product.

**Table 1:** Primers used in this study for mutant construction, confirmation and sequencing. Primers used in the amplification of the homology arms and SOE_PCR were designed using D39 genome as reference, primers designed later to sequence the mutants were designed using 519/43 genome data.

Primer name	Sequence (5′-3′)	Gene
plyFw1_ NotI	TTT GCGGCCGCCAGTAAATGACTTTATACTAGCTATG	*ply*
plyRv1_BamHI	CGAAATATAGACCAAAGGACGCGGATCCAGAACCAAA CTTGACCTTGA	*ply*
plyFw2_BamHI	TCAAGGTCAAGTTTGGTTCTGGATCCGCGTCCTTTGGTCTATATTTCG	*ply*
plyRv2_NotI	TTTGCGGCCGCCATTTTCTACCTTATCCTCTACC	*ply*
BamHI_SP2F2	GGATCC CTA GAA CTA GTG GAT CCC CC	spc cassette from pR412
BamHI_SP2R2	GGATCC AAT TCT GCA GAT TTT AC ATG ATC	spc cassette from pR412
SPEC_REV	TAATTCCTCTAAGTCATAATTTCCG	spc cassette from pR412
plySCN1	CCAATGGAAATCGCTAGGCAAGAGATAA	+/1602753
plySCN2	ATTACTTAGTCCAACCACGGCTGAT	SWT_01592
spec_sqr1	CCTGATCCAAACATGTAAGTACC	spc cassette from pR412
spec_sqf2	CGTAGTTATCTTGGAGAGAATA	spc cassette from pR412
spec_sqf1	GGTACTTACATGTTTGGATCAGG	spc cassette from pR412
spec_sqr2	TATTCTCTCCAAGATAACTACG	spc cassette from pR412

The spectinomycin cassette from plasmid pR412 (Dr Marc PrudHomme CNRS-Universite Paul Sabatier Toulouse France) was amplified using primers BamHI_SP2F2 and BamHI_SP2R2 (Table 1). The PCR product was excised and purified as described above.

### 2.3 Construction of plasmid pSD1 and transformation of *E. coli* Dh5α

A ligation reaction was performed following the pGEMT-easy system I manufacturer instructions (Promega, UK). Briefly, 5 μl 2X ligation buffer, 1 μl pGEMT-easy, 2 μl of the *ply*_SOE product, 1 μl T4 DNA ligase and water were combined in a microcentrifuge tube and incubated overnight at 4 °C. This generated plasmid pSD1.

Chemically competent *E. coli* Dh5α (Invitrogen UK) were transformed with pSD1. Briefly, 50 μl of *E. coli* Dh5α were incubated with 3 μl ligation reaction for 15 min on ice followed by thermic shock (42 °C, 30 s). The cells were then placed on ice for 2 min. SOC media was added and the cells recovered for 2 h at 37 °C. The transformation was then plated on Luria Bertani Agar (LBA) and blue/white selection used to discern the transformants carrying pSD1.

### 2.4 Extraction and restriction digestion of pSD1 and spectinomycin gene and construction of pSD2

Plasmid DNA extraction was performed according to the manufacturer's instructions. (NEB, Monarch, UK). 1 μg of pSD1 and spectinomycin cassette were digested with BamHI at 37 °C for 3 h. The restriction digest was analysed by electrophoresis, excised and purified. A ligation reaction was prepared using BamHI- digested pSD1 and spectinomycin, following the pGEMT-easy system I manufacturer instructions (Promega, UK). Briefly, 5 μl 2X ligation buffer, 2 μl pSD1, 2 μl of spectinomycin cassette, 1 μl T4 DNA ligase and water were combined in a microcentrifuge tube and incubated overnight at 4 °C. This generated plasmid pSD2. Plasmid pSD2 was transformed into *E. coli* Dh5α as described above. Transformants carrying plasmid pSD2 were selected based on their ability to grow in LBA supplemented with 100 μg/ml of spectinomycin and ampicillin.

### 2.5 Transformation of *S. pneumoniae* strain 519/43

*S. pneumoniae* strain 519/43, ST 5316, serotype 1 was transformed with 500 ng of pSD2. Briefly, *S. pneumoniae* 519/43 was grown overnight in BHI at 37 °C, 5% CO_2_. In the morning the cultures were diluted 1:50 and 1:100 in 10 ml of fresh BHI broth (Oxoid, UK). The bacterial suspension was incubated at 37 °C until the OD_595nm_ was 0.05–0.1. At this stage 860 μl were taken and put into a clear microcentrifuge tube and 100 μl 100 mM NaOH, 10 μl 20% (w/v) BSA, 10 μl 100 mM CaCl_2_ and 2 μl 50 ng/ml CSP1 [32] were added. The reaction was then incubated at 37 °C, statically for 3 h 330 μl were plated onto Blood Agar (BA) plates supplemented with 100 μg/ml spectinomycin, every hour over the 3 incubation hours. The plates were incubated overnight at 37 °C, 5% CO_2_. Spectinomycin resistant colonies were patched onto another BA plate supplemented with 100 μg/ml spectinomycin. They were also plated in BA supplemented with 100 μg/ml ampicillin to test for the presence of the plasmid backbone. Presence of the spectinomycin cassette was determined by PCR using primers plyFw1_ NOTI and SPEC_REV and confirmation of the mutation was performed using primers plySCN1 and plySCN2 (Table 1).

### 2.6 Phenotypic characterisation

#### 2.6.1 Assessment of 519/43Δply ability to grow in BHI

Growth impairment was assessed by growth curves. 100 μl of isogenic mutant 519/43Δply and wild type 519/43 were inoculated into 10 ml BHI broth (Oxoid, UK). The cultures were grown at 37 °C statically, in an incubator with 5% CO_2_ for 24 h. Optical density (OD) was read at 600 nm every 2 h from time 0 h to time 8 h, and then one last measurement was taken at 24 h.

#### 2.6.2 Assessment of 519/43Δply ability to grow in serum

Wild-type and *ply*-deleted *S. pneumoniae* (519/43) were grown in Todd-Hewitt broth supplemented with 0.5% yeast extract (THY) to mid-log phase at 37 °C in 5% CO_2_. Bacterial density was calculated by counting the colony forming units (CFU) in 100 μl of ten-fold serially diluted stocks on Colombia agar supplemented with horse blood after 24 h. Stocks of each isolate were made in 80% glycerol and stored at −80 °C. Stocks were thawed, washed and re-suspended at a starting bacterial count of 1 × 10^5^ CFU in healthy donor human serum. Each sample was cultured in triplicate in 100 μl volume at 37 °C in 5% CO_2_ for 24 h in the Tecan Spark® microplate reader (Tecan, USA). Optical density (OD) readings at 600 nm were taken every 30 min. Data were plotted using Prism version 7.

#### 2.6.3 Expression of Pneumolysin in 519/43 WT and 519/43Δ*ply*

Expression of Pneumolysin was assessed by Western blot. Briefly, bacterial cultures were grown overnight at 37 °C, statically. The next day, 1 ml of culture was taken spun down at 5000×*g*. The pellet was resuspended in 50 μl of NuPage LDS Sample buffer (4X) (Thermo Fisher Scientific, UK) and the samples boiled for 10 min 50 μl of supernatant were also taken from the overnight grown cultures. 10 μl of the cell lysates and supernatants of both 519/43 WT and 519/43Δ*ply* were run in a NU-PAGE 4–12% Bis-Tris Protein Gel (Thermo Fisher Scientific, UK) and blotted onto a PVDF membrane using a iBlot 2 (Thermo Fisher Scientific, UK). The presence of Pneumolysin was assessed using Mouse Anti-Pneumolysin antibody [PLY-4] (abcam, UK) and detected using IRDye®680RD Goat anti-mouse IgG (H + L).

#### 2.6.4 Haemolysis assessment

Supernatant of pneumococcal bacterial cultures from 519/43, 519/43Δ*ply* and D39 were mixed with a 5% solution of horse blood in Alseevers (RBCs) in PBS and incubated in triplicate at 37 °C for 30 min in 96-well round-bottom plates. The plates were centrifuged at 2300×*g* for 5 min to pellet non lysed RBCs. The supernatants were carefully transferred to another 96-well round bottom plate and the supernatants were measured for haemoglobin content by spectrophotometry at A450. Saponin 0.5% was used as the positive control representing 100% haemolysis. Haemolysis percentages were determined by averaging the absorbance values for each sample and converting them to a percent value when compared to the positive control (Saponin 0.5%).

#### 2.6.5 In vivo virulence of 519/43 Δ*ply*

Mice (5-6-week-old CD1, Charles River) were anaesthetised with isoflurane then given 50 μl of bacteria suspended in PBS intranasally by pipetting onto the nares (Dose 519/43: 3.4 × 10^7^, 519/43Δply: 5.0 × 10^7^). Alternatively, mice were injected with 100 μl of bacterial suspension intraperitoneally (dose 519/43: 3.45 × 10^4^; 519/43Δply: 3.8 × 10^4^) without anaesthesia. The challenge dose was confirmed by retrospectively diluting and plating the culture on blood agar. Mice were euthanized at the pre-determined time points (24h post infection) by CO_2_ asphyxiation. Blood was collected from the aorta. Following perfusion with PBS, the lungs were excised. These samples were homogenised in 1 ml PBS using a Precellys® 24 tissue homogeniser [Bertin Technologies, France]. Samples of the blood and lungs were then serially diluted in serum broth and colonies were enumerated after overnight growth on blood agar plates supplemented with spectinomycin when appropriate.

## 3 Results

### 3.1 Generation of plasmid pSD1

**Fig. 1:**
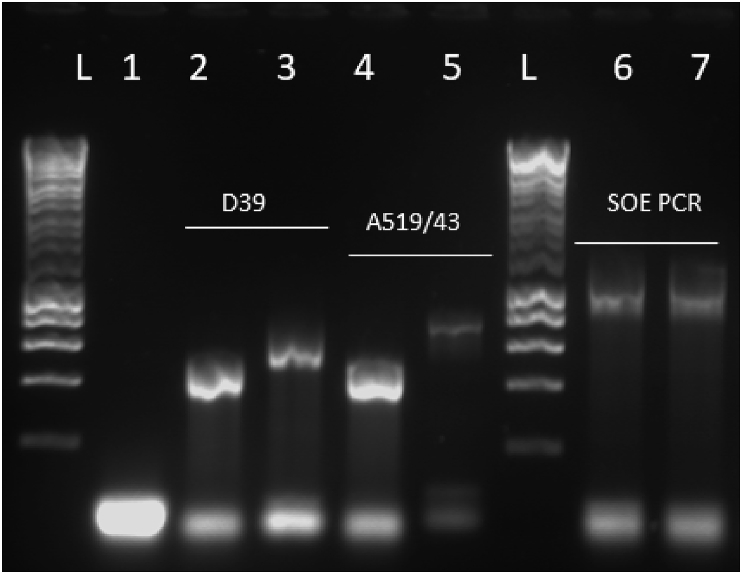
Amplification of homology arms (left hand side of the figure); and Splicing by Overlapping Extension PCR (right hand side of the figure). L-ladder, lane 1- negative control for the reaction, lane 2 and 3- left (527 bp) and right (756 bp) homology arm amplified from D39 gDNA, lanes 4 and 5- left (527 bp) and right (756 bp) homology arms amplified from 519/43 gDNA. Right hand side lane 6- D39 SOE PCR product, lane 7–519/43 SOE PCR product (1235 bp).

A pneumolysin mutant was made in the 519/43 serotype 1 *S. pneumoniae* strain as described in the Material and Methods section. Firstly, upstream and downstream homology arms were amplified using DNA from both D39 (serotype 2) and 519/43 (serotype 1) Fig. 1, lanes 2 and 3, 4 and 5 respectively. At the time that this mutant was constructed strain 519/43 was not sequenced. Therefore, to ensure the primers were functional, D39 genomic DNA was used as a positive control for the binding. Secondly, SOE-PCR was performed to fuse the right and left DNA arms as a 1235 bp product (Fig. 1, lanes 6 and 7) whilst introducing a BamHI site at the junction of upstream and downstream DNA arms. The 1235bp product was TA-cloned into pGEM-Teasy. This generated plasmid pSD1.

**Fig. 2:**
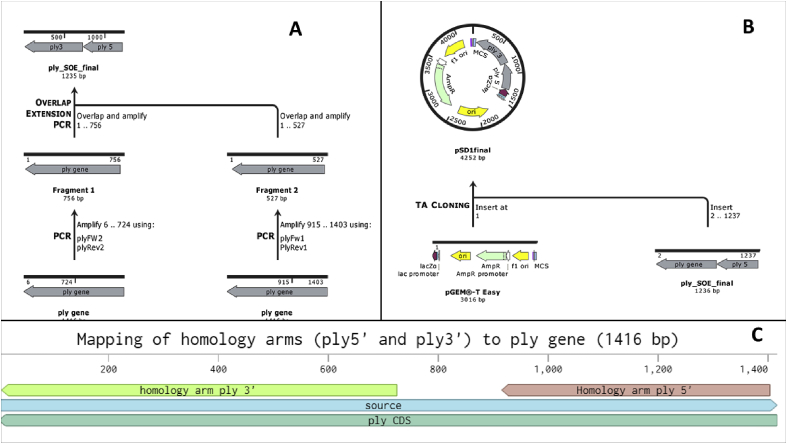
A Scheme depicting the strategy used for SOE-PCR (A). B- Depicting how pSD1 was generated. C- Mapping of the homology arms to the pneumolysin gene.

### 3.2 Generation of plasmid pSD2

**Fig. 3:**
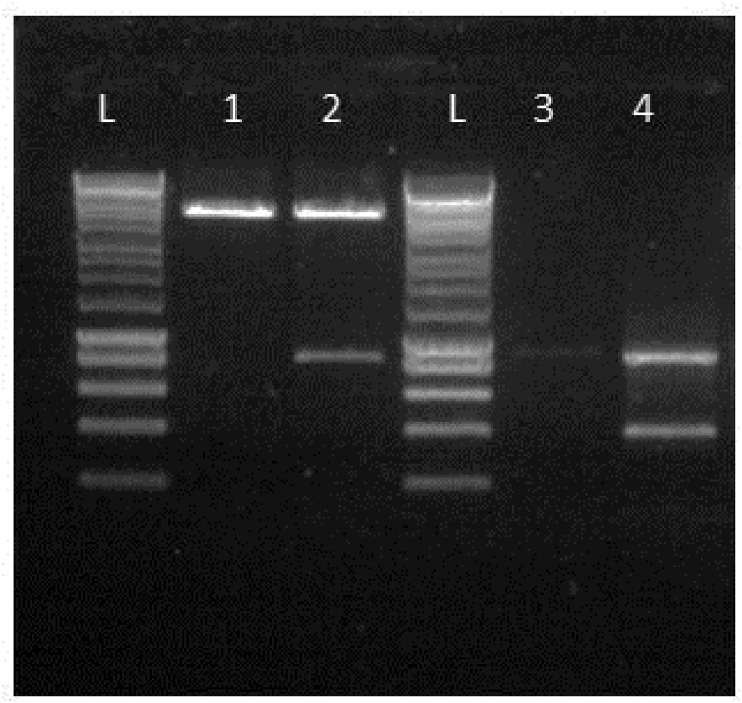
Confirmation of presence of spectinomycin cassette in pSD2. L-hyperladder 1 kb (Bioline); 1- pSD1 digested with BamHI; 2-pSD2 digested with BamHI; L hyperladder 1 kb; 3- Spectinomycin cassette amplified from pR412, 4- Spectinomycin cassette digested with BamHI.

Plasmid pSD2 containing a suitable antibiotic resistance cassette between the 5′ and 3’ arms of the sequences flanking *ply* was created by BamHI digestion of plasmid pSD1 (Fig. 3, lane 1) and ligation to a previously digested spectinomycin cassette (Fig. 3, lane 4) as described above. The insertion of the spectinomycin cassette was at the junction of the upstream and downstream homology arms where the BamHI site was introduced at the time of the PCR. Strain 519/43 was then transformed with 500 ng of plasmid pSD2 using CSP1 to induce competence. The transformation yielded 10 colonies. 7 out of the 10 were positive for the spectinomycin insertion confirmed by PCR.

**Fig. 4:**
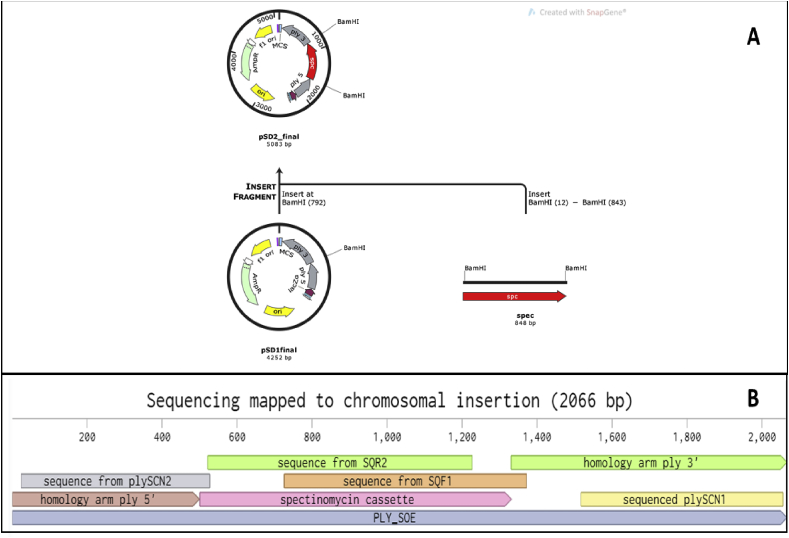
(A) Scheme showing the assembly of plasmid pSD2. (B) Mapping of the sequencing data to the chromosomally inserted ply_spec

The mutation by insertion of the spectinomycin cassette on 519/43 genome was confirmed in two transformants by sequencing with primers *spec_sqr1, spec_sqf2, spec_sqf1* and *spec_sqr2,* plySCN1 and plySCN2 (Table 1) (Fig. 4B). Primers plySCN1 and plySCN2 (Table 1) were designed with attachment site outside of the homology zone to confirm the insertion of the spectinomycin cassette in the chromosome.

### 3.3 Growth of the *Δply* mutants in rich media and in healthy donor serum

**Fig. 5:**
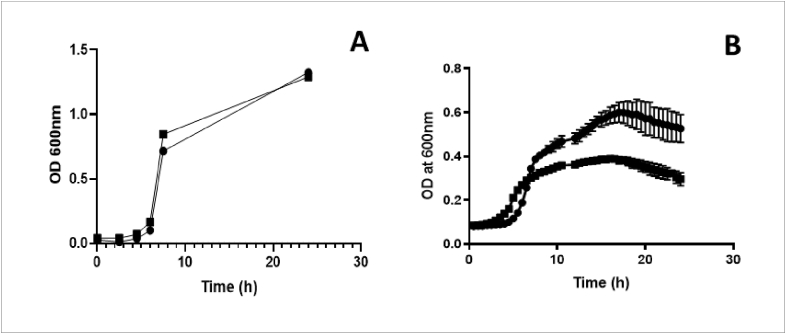
(A) Growth of 519/43WT and 519/43Δply in BHI (Oxoid, UK) over 24h. ● represents 519/43WT and ▪ represents 519/43 Δply. For each time point there are three technical and two biological replicates (B) Growth of 519/43WT and 519/43Δply in healthy donor serum over 24 h ● represents 519/43WT and ▪ represents 519/43 Δply. For each time point there are three technical repeats.

Mutations by insertion can on occasion cause growth defects. To ascertain that this was not the case isogenic *Δply* mutants and wild type strain were cultured in BHI (rich media) and growth monitored using optical density. There were no significant differences in growth between the isogenic mutant 519/43Δ*ply* and the wild type 519/43 strains, demonstrating that the insertion did not cause any noticeable growth defect in rich media (Fig. 5A). On the other hand, there were significant differences when 519/43WT and 519/43Δ*ply* were grown in serum. Where the wild type strain was able to continue growing exponentially the same wasn't observed for the mutant. The mutant grew at similar rate until 7 h when the growth defect became noticeable. From then until the end of the experiment (24 h) 519/43Δ*ply* continued to grow but at a much lower rate when compared with the wild type (p < 0.05) (Fig. 5B).

### 3.4 Expression of Pneumolysin in 519/43 WT and 519/43Δ*ply*

**Fig. 6:**
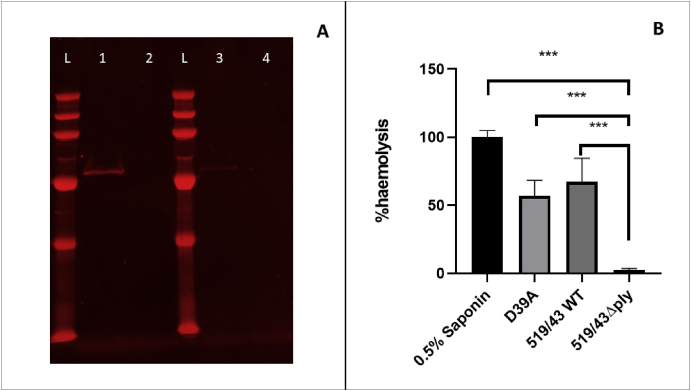
A- Expression of Pneumolysin in 519/43WT and isogenic mutant 519/43Δply. L- PageRuler Plus prestained protein ladder, 1- Pneumolysin present in cell lysate of 519/43WT, 2- Absence of Pneumolysin in 519/43Δply cell lysate; 3- Presence of Pneumolysin in 519/43WT supernatant; 4- Absence of Pneumolysin in 519/43Δply supernatant. B- Determination of haemolytic activity of D39, 519/43WT and isogenic mutant 519/43Δply, when compared to 0.5% saponin. Saponin-derived haemolysis is considered 100% and the values for 519/43 wt and 519/43Δply were calculated against this. Each value plotted is the mean of 5 technical and 3 biological replicates.

The expression of Pneumolysin was assessed by western blot to confirm that the protein was no longer present as it could still have a cytotoxic effect. It was clear that Pneumolysin was not present on 519/43Δ*ply* in either the cell lysate or in the supernatant but it was present in the wild type (Fig. 6A).

### 3.5 Haemolysis assessment

Pneumolysin is responsible for red blood cell lysis by *S. pneumoniae*. Hence, a haemolysis assay was used to confirm that the mutation had rendered pneumolysin non-functional. Saponin was used as the positive control (100% haemolysis after 30 min incubation), and percentage values for all samples were calculated against saponin. In comparison to saponin, 519/43WT lysed 67.3% red blood cells (RBCs) in a 30 min incubation period; by contrast the defined 519/43*Δply* mutant lysed only 2.5% of the cells (Fig. 6B). As the pneumolysin allele present in 519/43WT is different to the D39 allele (it contains one amino acid substitution, D380 N) the haemolysis of the D39 strains were also assessed. This one amino acid substitution is often observed on ST227 strains [33] and does not seem to confer a haemolytic advantage. D39 pneumolysin had an activity of 56.8% when compared to saponin control, a lower activity than that recorded for 519/43 WT although the difference was not statistically significant. The data confirm that the insertion of the spectinomycin cassette has disrupted the ability of pneumolysin to lyse RBCs.

### 3.6 Role of 519/43 pneumolysin in infection

**Fig. 7:**
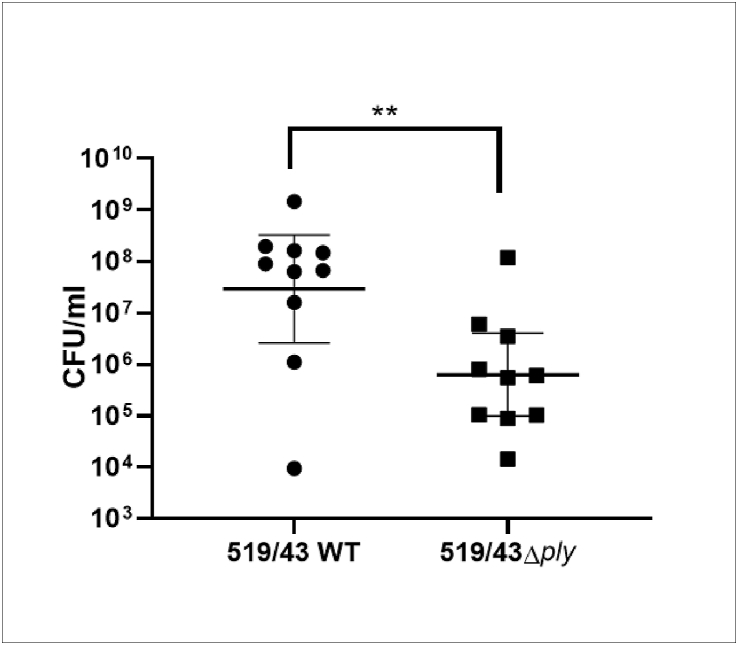
Intraperitoneal infection of CD1 mice. Each dot represents 1 mouse. Presented is the geometric mean with 95% CI. Mice were euthanized humanely at 24 h post infection and the cfu in blood enumerated.

Due to the known multiple roles of pneumolysin on virulence and since some serotype 1 strains (ST306) express a non-haemolytic pneumolysin [34], the contribution of this protein to infection in strain 519/43 was assessed. Outbred CD1 mice were infected intraperitoneally with 519/43 (3.5 × 10^4^ cfu/ml) and 519/43Δ*ply* (3.8 × 10^4^ cfu/ml). The data showed that after 24 h there was a statistically significant difference in the number of pneumococci present in blood with 2.9 × 10^7^ cfu/ml of the 519/43 strain and 6.2 × 10^5^ cfu/ml of the 519/43Δply strain (p < 0.01, n = 10), suggesting a role for pneumolysin during systemic infection (Fig. 7).

**Fig. 8:**
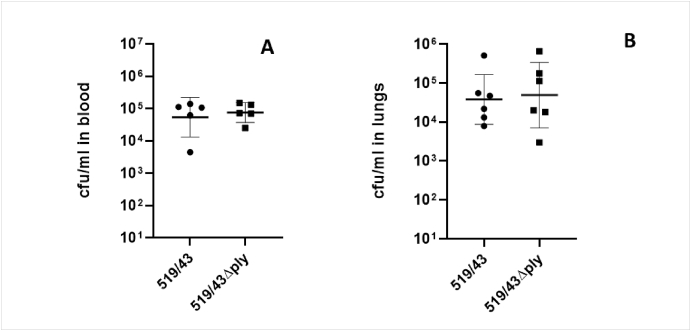
Intranasal infection of CD1 mice. Each dot represents 1 mouse. (A) Cfu/ml present in blood after intranasal infection. (B) cfu/ml present in the lungs after intranasal infection. Presented is the geometric mean with 95% CI. Mice were euthanized humanely at 24h post infection and the cfu in blood and lungs enumerated.

However, the same trend wasn't observed for the pneumonia models. When mice were infected with 519/43 (3.4 × 10^7^ cfu/ml) and 519/43Δply (5.0 × 10^7^ cfu/ml) intranasally there were no significant differences between cfu present in blood (519/43WT: 5.4 × 10^4^ cfu/ml and 519/43Δply: 7.6 × 10^4^ cfu/ml) or in the lungs (519/43: 3.8 × 10^4^ cfu/ml) and 519/43Δply: 4.9 × 10^4^ cfu/ml) between the wild type and the isogenic mutant after 24h of infection (Fig. 8).

## 4 Discussion

*Streptococcus pneumoniae*, particularly serotype 1, continues to pose a threat in most low- and middle-income countries. Despite the introduction of the vaccines PCV7, PCV10 and more recently PCV13 that include polysaccharide in their formulation to protect against serotype 1, this important serotype is still capable of causing outbreaks that lead to high mortality and morbidity. Because of its clinical importance, the ability to genetically manipulate serotype 1 isolates is paramount but has not, to our knowledge, previously been described.

In this study we report a serotype 1 strain 519/43 (ST5316), a clinical isolate from a meningitis patient in Denmark, that we have genetically manipulated to the same extent as other serotypes e.g. strains D39 (serotype 2) or TIGR4 (serotype 4). The success of our endeavour relies entirely on the choice of strain and on the transformation of 519/43 with a plasmid instead of linear DNA (the conventional methodology in *S. pneumoniae* mutagenesis); All attempts using linear DNA were unsuccessful. The reason why this strain can be genetically modified and other serotype 1 strains cannot, remain unknown. Genome sequences suggest that all natural competence genes in 519/43 are intact, whereas this is not true for all sequenced serotype 1 strains in which, for example, *comD* can be truncated or *comE* absent [35]. Despite this strain not being an important African sequence type, it was isolated from a meningitis patient making it a good candidate strain to study and understand the development of pneumococcal meningitis.

In order to show that this strain can be mutated, an insertion mutation was made in the pneumolysin gene. This gene was targeted as it has a readily identifiable phenotype *in vitro*, but we have also used the same approach to make mutations affecting additional genes in the serotype 1 strain 519/43 (ST5316) (manuscript in preparation).

We demonstrated that strain 519/43 has a haemolytic pneumolysin and that its function can be interrupted by inserting an antibiotic marker in its place. We also observed that the one amino acid substitution (D380N) common to our strain and to strains from ST227 [34] does not confer a higher ability to lyse RBCs *in vitro*. We also showed that pneumolysin mutation does not seem to impact bacterial growth in complete laboratory media, whereas this mutant had impaired growth in serum compared to the wild type strain. We showed that the haemolytic pneumolysin carried by 519/43 has an apparent role in invasive disease as we found significantly fewer bacteria in the blood of mice when challenged intraperitoneally with the loss of function mutant 519/43Δ*ply*. Nevertheless, although there were significantly fewer bacteria in blood at 24 h post infection, invasive disease still occurred and consequently the mice displayed visible signs of disease. A similar phenotype was observed for the pneumolysin deficient mutant of D39 [29,36]. In the past it was thought that pneumolysin was essential for IPD, but our data suggests that this is not the case for serotype 1 *S. pneumoniae*. Further support for this is provided by previous publications; Kirkham et al., demonstrated that a strain carrying a naturally occurring non-haemolytic pneumolysin did not prevent ST306 strains from causing invasive disease when infected intraperitoneally with ST306 or ST227 presenting with bacteraemia within 24 h of infection. Additionally Jefferies et al. reported that a clinical strain S1-11 (ST228) that is unable to express pneumolysin allele 14 due to the insertion of a mobile element within its gene was isolated from the blood of a patient with pneumonia, suggesting this strain could still cause disease and progress from the lungs to the blood [33].

In our study we observed that when mice were infected intranasally, there was no significant differences in bacterial load in blood and lungs between the 519/43 wild type and Δ*ply* strains. Similar results were observed by Kirkham et al. when they compared intranasal challenge by 01–2696 (ST227) that possesses a haemolytic pneumolysin with 01–1956 (ST306) that has a non-haemolytic pneumolysin; they observed no difference in bacterial loads and all mice were bacteraemic after 24 h. Conversely, Paton et al., showed that a sequence type associated with an outbreak carried a reduced haemolytic activity *ply* allele, and that mice infected with D39 carrying this reduced haemolysis allele for pneumolysin survived about 4 h longer than mice infected with D39. This was clearly different for mice infected with D39Δ*ply*, who survived 8 h longer than their wild type counterpart. Importantly, the studies described by Paton et al. were done by transferring the pneumolysin from serotype 1 into D39 due to difficulties with mutagenesis in the serotype 1 strain. This could have had a significant impact on the observations reported and highlights the importance of making mutations in the correct strain background as in our study. Nevertheless, there seems to be a high degree of variation on the role and contribution of pneumolysin for IPD depending on serotype, and perhaps even sequence type [29]. In our study we were unable to discern which animals survived the longest as the experiment had a pre-defined ending time point. These data suggest that, as other reports for serotype 1 strains [34], strain 519/43 is not dependent on haemolytic pneumolysin to cause disease. Further experiments are necessary to thoroughly understand the role of serotype 1 pneumolysin in invasive disease.

Research is undergoing to expand which serotype 1 strains can be transformed and therefore allowing the construction of targeted mutations in genes of interest. Finally, the focus of this study was to provide the scientific community with a serotype 1 strain that can be genetically manipulated allowing many questions to be answered especially elucidating the role of determinants involved directly in survival, colonisation and disease, particularly in relation to meningitis.

## CRediT authorship contribution statement

**Vanessa S. Terra:** Conceptualization, Data curation, Methodology, Validation, Writing - original draft, Funding acquisition, Writing - review & editing. **Charles D. Plumptre:** Data curation, Writing - review & editing. **Emma C. Wall:** Data curation, Writing - review & editing. **Jeremy S. Brown:** Supervision, Funding acquisition, Writing - review & editing. **Brendan W. Wren:** Supervision, Funding acquisition, Writing - review & editing.
